# Supplementary material for: Full-Length Transcriptomic Sequencing and Temporal Transcriptome Expression Profiling Analyses Offer Insights into Terpenoid Biosynthesis in Artemisia argyi
Source: Molecules. 2022 Sep 13;27(18):5948. doi: 10.3390/molecules27185948 (PMC9501300; doi:10.3390/molecules27185948)
Supplement: Supplementary file 1 [file molecules-27-05948-s001.zip › molecules-1759595-supplementary/Supplementary material/Supplementary material -Figure1-3.pdf]

Compounds 1: o-Cymene; Formula: C10H14

Index:XMW0127; Rt=10.032min

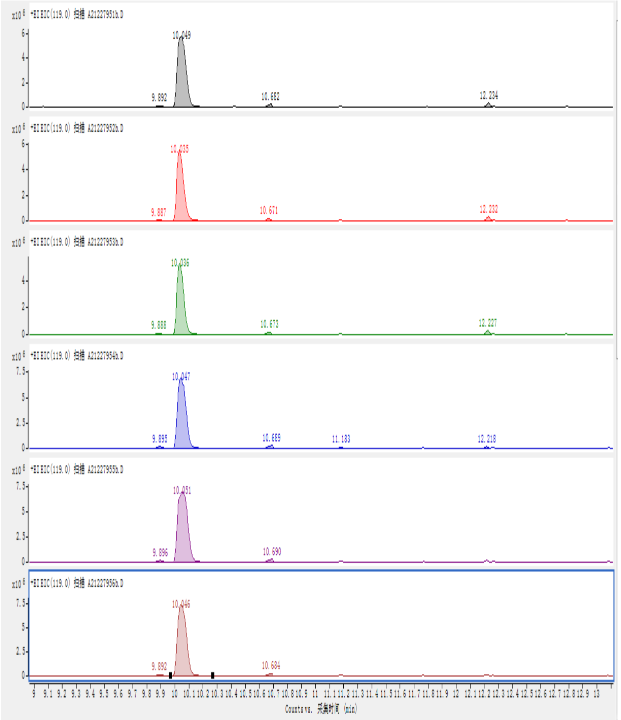

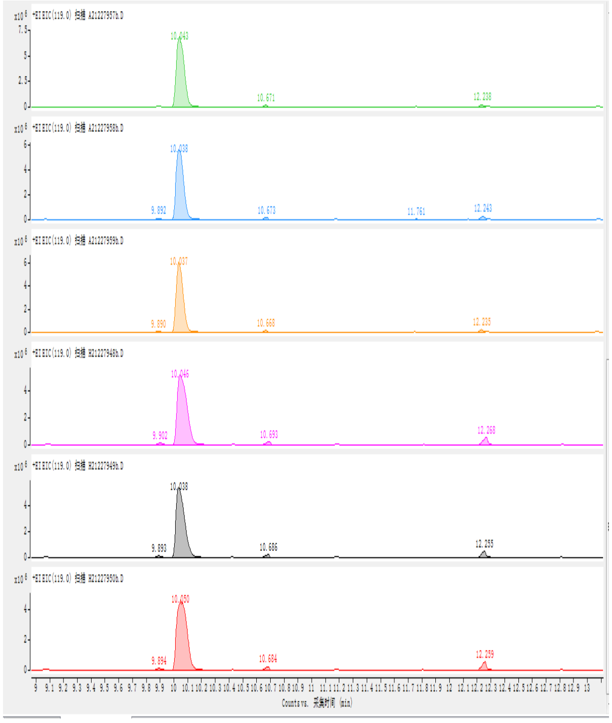

Compounds 2: 1,8-Cineole ; Formula: C10H18O

Index:KMW0218; Rt=10.2449

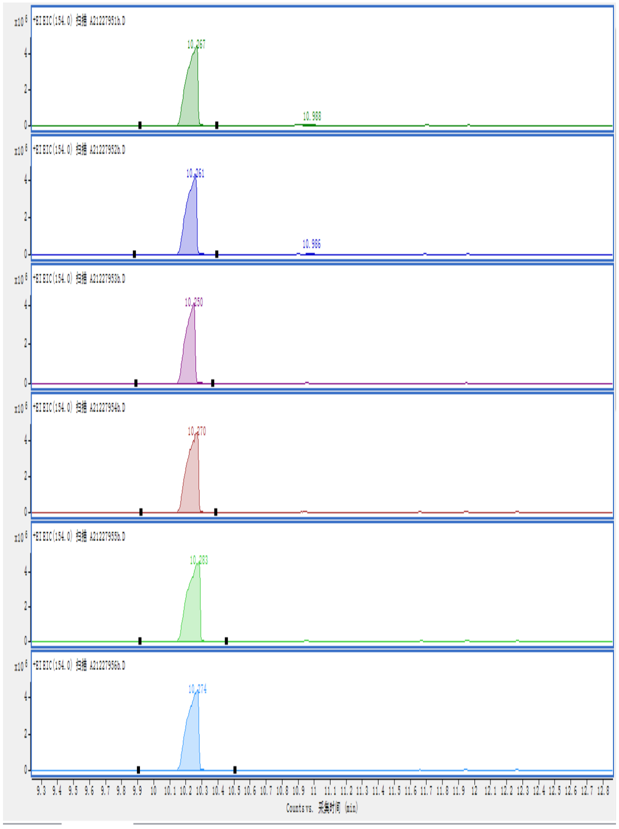

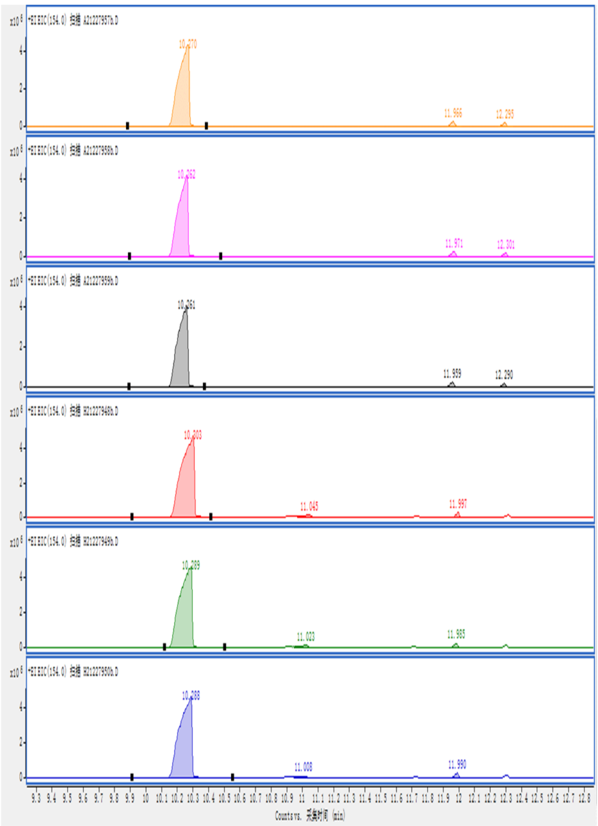

Compounds 3:(1R)-4-methyl-1-propan; Formula: C10H18O

Index:NMW0005; Rt=13.0314

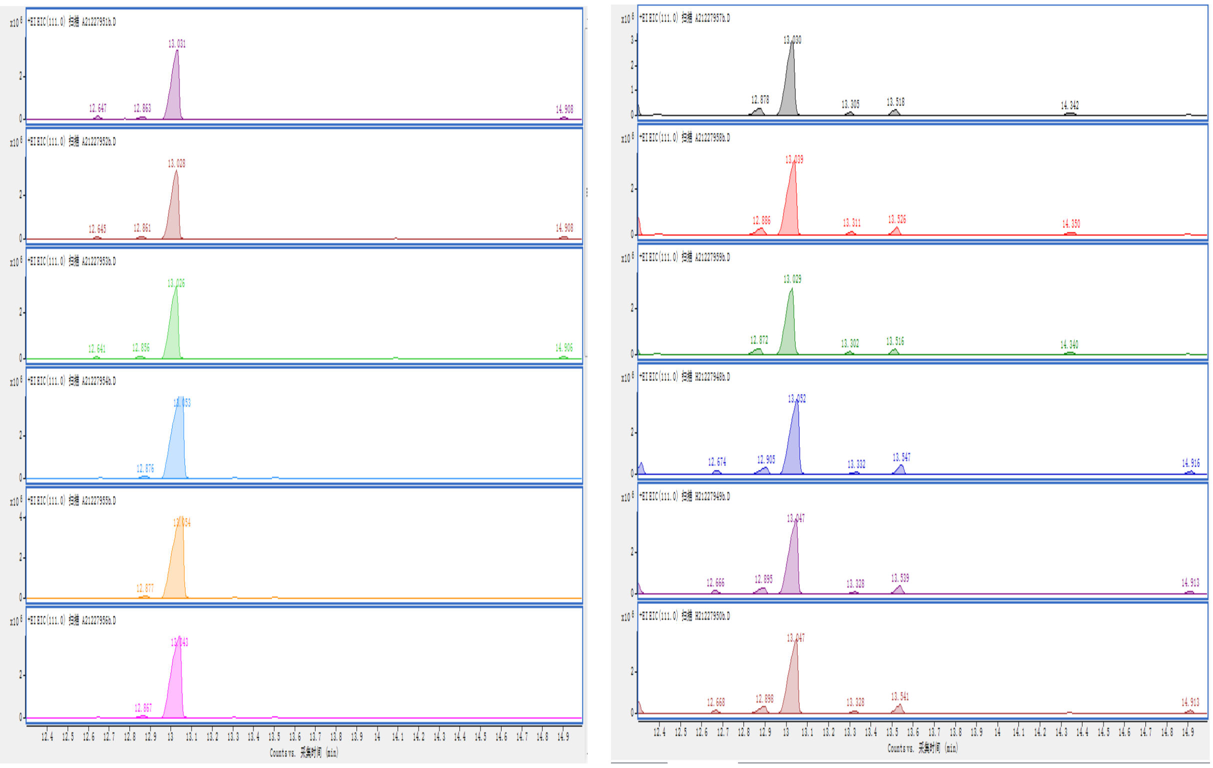

Compounds 4: γ--Terpinene; Formula: C10H16

Index:KMW0259; Rt=10.6687

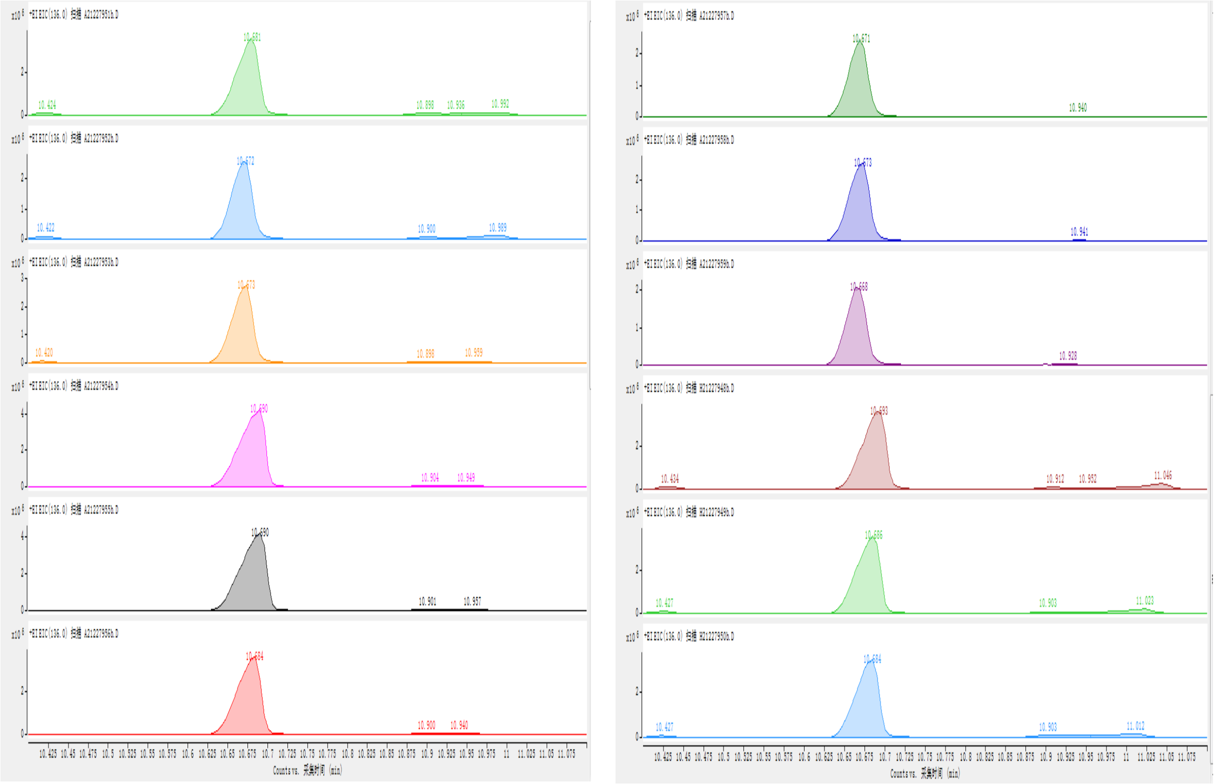

Compounds 5: β-Caryophyllene; Formula: C15H24

Index:KMW0565; Rt=17.5135

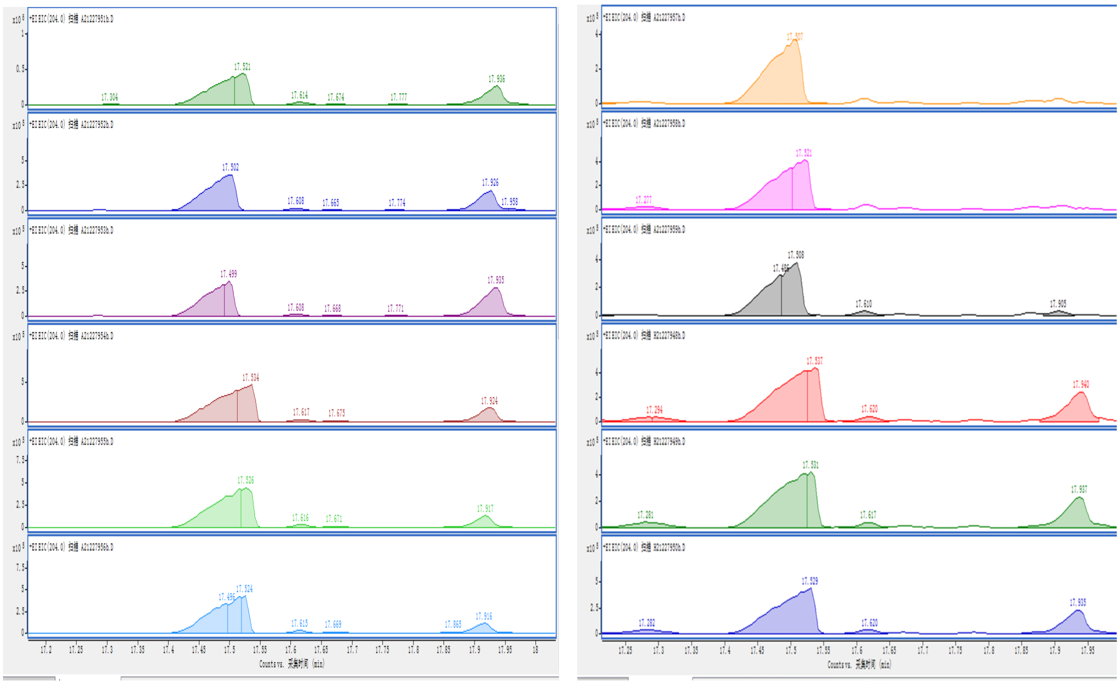

Compounds 6: Sabinene; Formula: C10H16

Index:KMW0178; Rt=9.0522

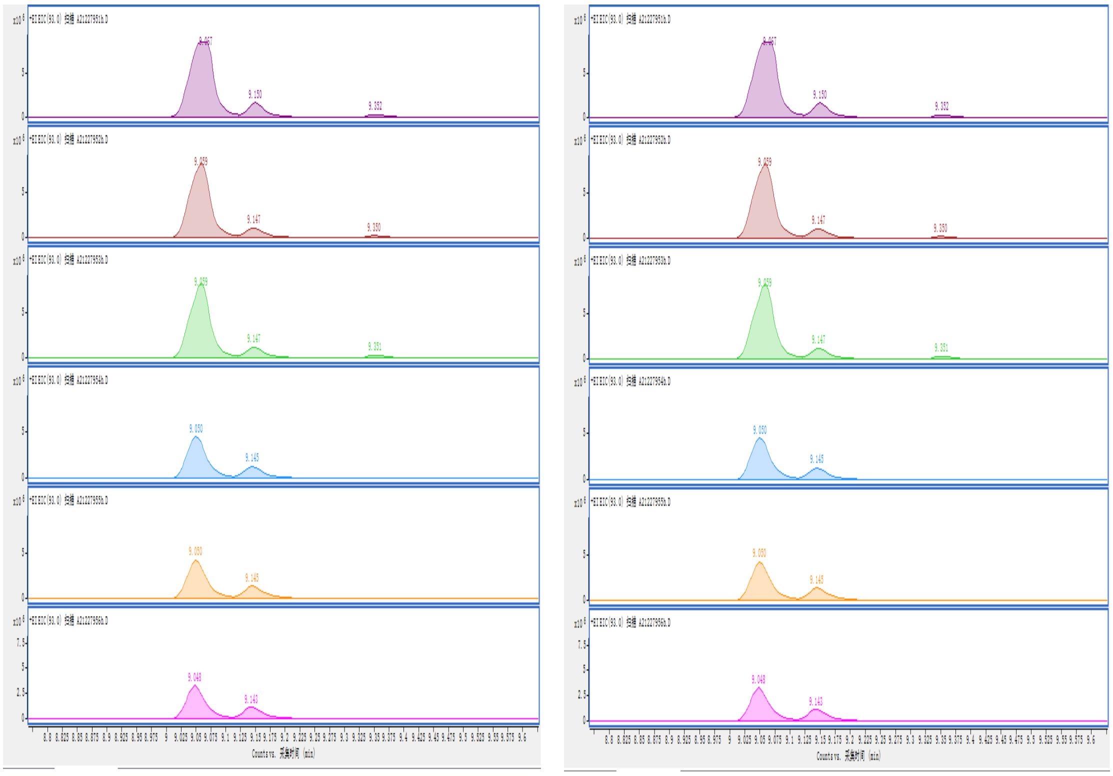

Compounds 7: Thujone; Formula: C10H16O

Index:WMW0194; Rt=11.8547

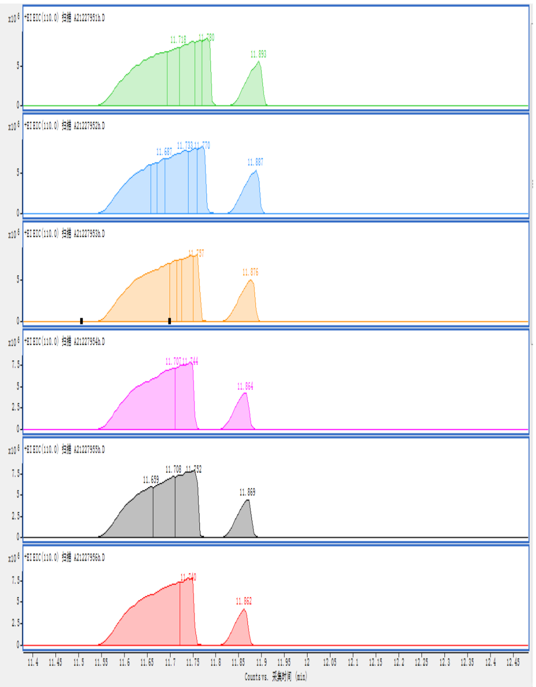

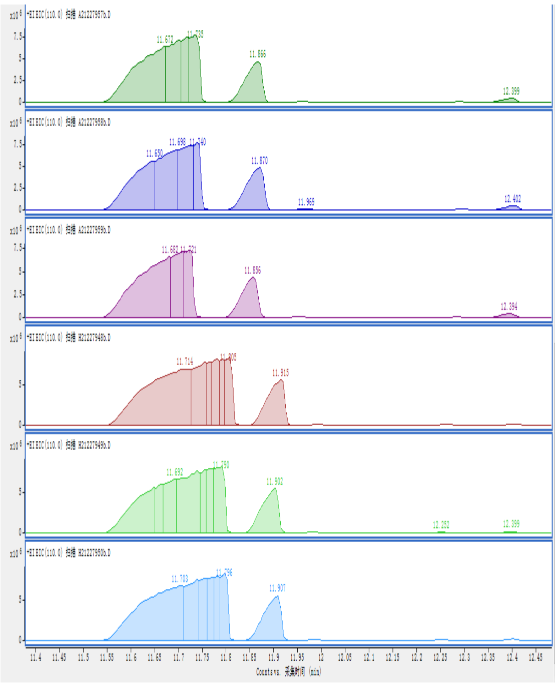

Compounds 8: β-Farnesene ; Formula: C15H24

Index:KMW0478; Rt=17.921

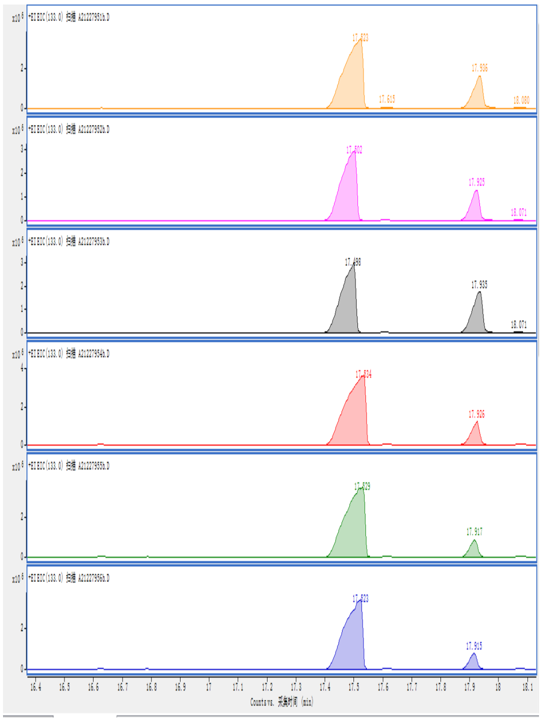

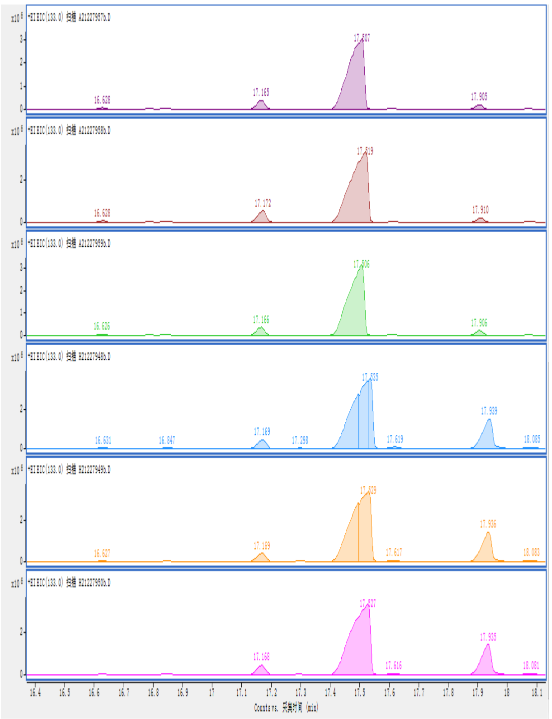

Compounds 9: α-Terpineol ; Formula: C10H18O

Index:NMW0071; Rt=13.2728

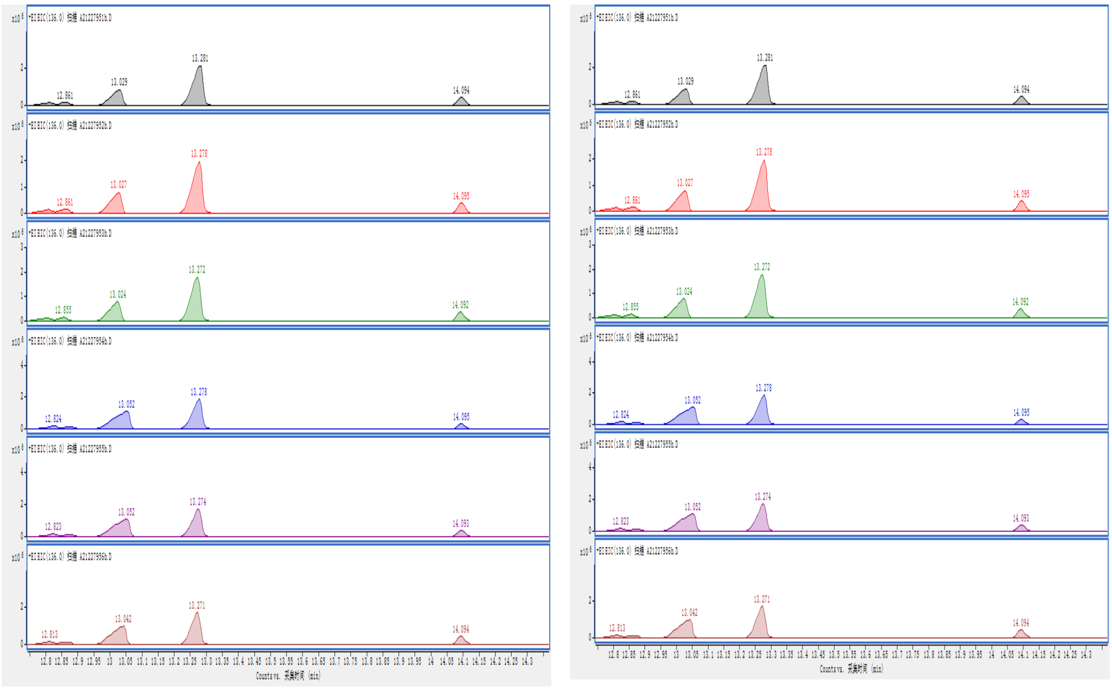

Compounds 10: β-Selinene; Formula: C15H24

Index:KMW0556; Rt=18.6734

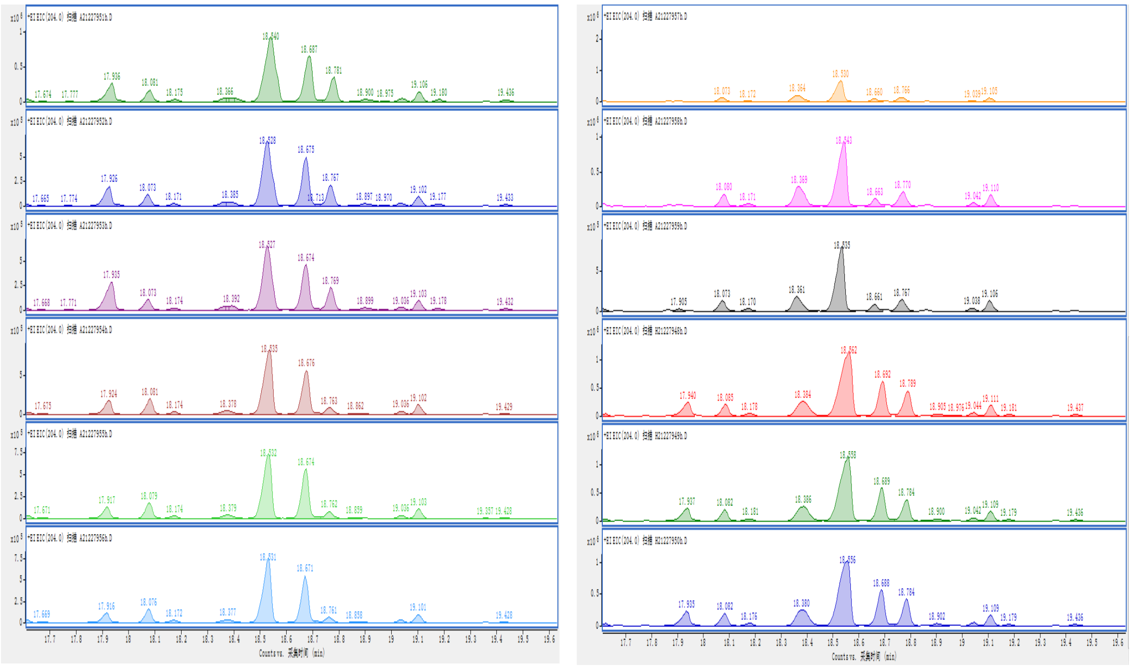

Figure S1. GC-MS chromatogram in extracted ion chromatogram (EIC) mode of 10 constituents in *Folium A. Argyi* extract.

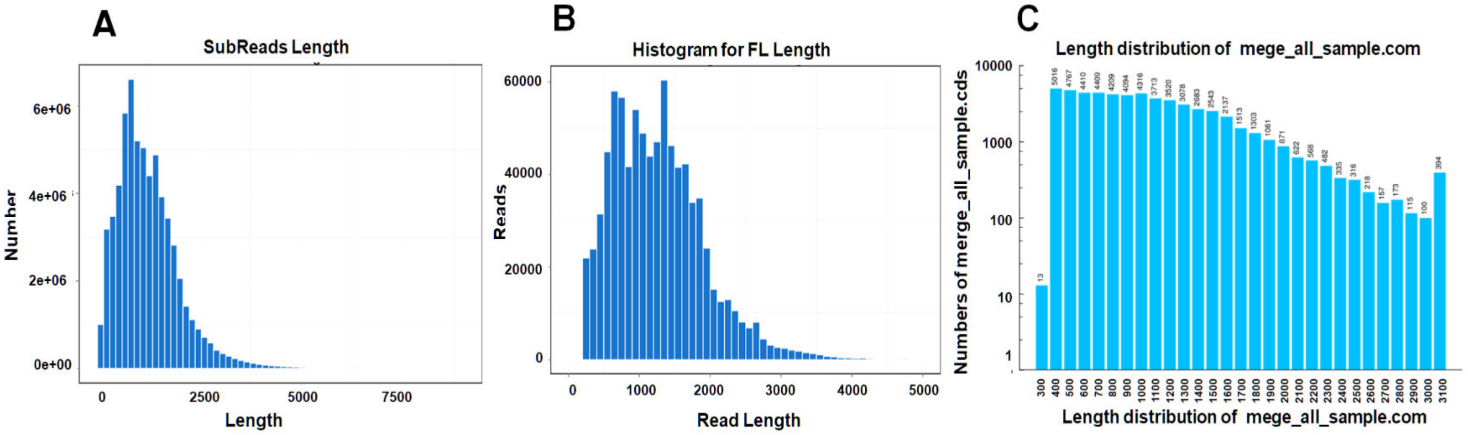

Figure S2. PacBio single-molecule long-read sequencing of *A. argyi*. (A) Subread length distribution. (B) Length distribution of full-length non-chimeric reads. (C) Length distribution of CDSs of all isoforms.

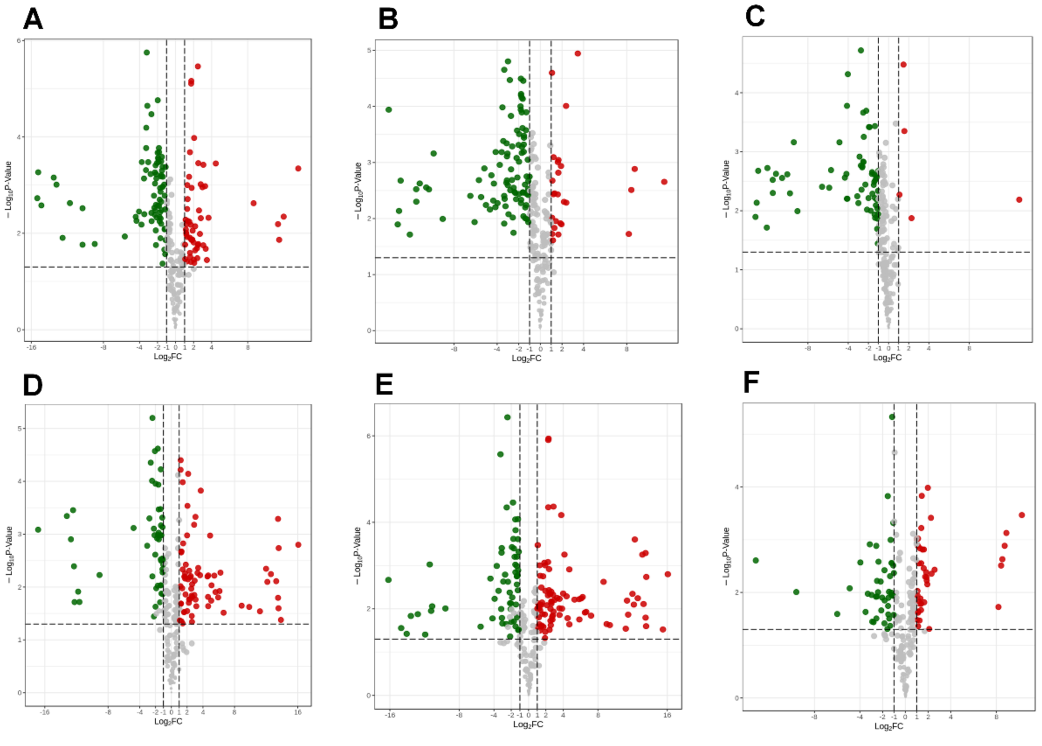

Figure S3. Volcano plots exhibiting differential expression of genes associated with different *A. argyi* developmental stages. (A) Samples harvested in April and July; (B) Samples harvested in April and June; (C) Samples harvested in April and May; (D) Samples harvested in June and July; (E) Samples harvested in May and July. (F) Samples harvested in May and June
