# Supplementary material for: Full-Length Transcriptomic Sequencing and Temporal Transcriptome Expression Profiling Analyses Offer Insights into Terpenoid Biosynthesis in Artemisia argyi
Source: Molecules. 2022 Sep 13;27(18):5948. doi: 10.3390/molecules27185948 (PMC9501300; doi:10.3390/molecules27185948)
Supplement: Supplementary file 1 [file molecules-27-05948-s001.zip › molecules-1759595-supplementary/Supplementary material/Supplementary material -Table2- 11.pdf]

Table S2 Statistics of polymerase read results

Library	Cell Number	Total Reads	Total Base(GB)	MaxLength(bp)	MeanLength(bp)	N50 Length(bp)
WHARTmjlEAAA0-5RAAPBI	1	1428605	84.81	336177	59367.63	118122

Table S3 Statistics of subread results

Sample	Library	Cell Number	Total Reads	Total Base(GB)	MaxLength(bp)	MeanLength(bp)	N50 Length(bp)
Qiai	WHARTmjlEAAA0-5RAAPBI	1	63281536	80.18	262660	1267.11	1571

Table S4 Statistics of CCS results

Sample	Library	Cell Number	Reads of Insert	Mean Read Length of Insert(bp)	Mean Read Quality of Insert	Mean Number of Passes
Qiai	WHARTmjlEAAA0-5RAAPBI	1	888748	1616	0.99	46

Table S5 PacBio single-molecular long-read sequencing of *A. argyi*

Sample	Library	Cell Number	Classifyed ROI	Full-length non-chimeric reads	Mean FL per ROI	Mean Read Quality	Mean Number of Passes	Mean full-length non-chimeric read length(bp)
Qiai	WHARTmjlEAAA0-5RAAPBI	1	740702	855813	1	0.99	42	1268

Table S6 Statistics of isoform results

Sample	Library	Cell Number	Total isoforms	Mean Quality	Mean isoforms length(bp)	Mean Full length coverage
Qiai	WHARTmjlEAAA0-5RAAPBI	1	241963	0.99	1395	Qiai

Table S7 The number of transcripts of *A. argyi*

Library	sLibrary_genes	[AllSamples]_genes
Qiai_WHARTmjlEAAA0-5RAAPBI	76,158	69,178
TOTAL	76,158	69,178

Table S8 Statistics of transcripts after redundant sequence removal

Total_number	Total_length	N50	N90	Max_length	Min_length	Sequence_GC(%)
69,178	92,392,860	1,619	795	5,657	200	40.71%

Table S9 Statistics of CDS prediction results

Total_number	Total_length	N50	N90	Max_length	Min_length	Sequence_GC(%)
53,136	59,241,828	1242	582	5125	297	43.02%

Table S10 Functional annotation and classification of assembled unigenes in *A. argyi*

Values	Total	Nr	Nt	Swissprot	KEGG	KOG	Pfam	GO	Intersection	Overall
Number	69,178	64,747	54,149	52,983	52,917	52,029	53,299	53,710	32,691	65,893
Percentage	100%	93.59%	78.27%	76.59%	76.49%	75.21%	77.05%	77.64%	47.26%	95.25%

Table S12 Primers for qRT-PCR

Primer	Sequence (5'- 3')
HMGR1_F	GACGATGGTAGCTCATGCGA
HMGR1_R	TTGTCAACCCATGGATGCCT
HDS7_F	TCGTGCCCATCTTGTGGTAG
HDS7_R	GGAGCACCACCGACATATCC
HDR1_F	CTCGCACAGACTACCTCTCC
HDR1_R	ATTGCGTTGACGATGACGTG
HDR3_F	CCACTCAGGAGCGACAAGAT
HDR3_R	TGTTGCTCGAGTTGAACCCA
TPS2_F	TGTAAAGGAGATGAAGATTTTGCTG
TPS2_R	GCATTTCCAGTAAACCCGTCAT
TPS5_F	CCTGTTGTCGCAGCATTCTCG
TPS5_R	ATCCATAGATCCTCCAACATCG
GAPDF-F	GACCAGAGGCTATTGGACGCT
GAPDH-R	ACATTAGGGGTGGGGACACGAAG
